# Supplementary material for: Myeloperoxidase enhances the migration and invasion of human choriocarcinoma JEG-3 cells
Source: Redox Biol. 2023 Sep 19;67:102885. doi: 10.1016/j.redox.2023.102885 (PMC10556814; doi:10.1016/j.redox.2023.102885)
Supplement: Multimedia component 1 [file mmc1.docx]

**
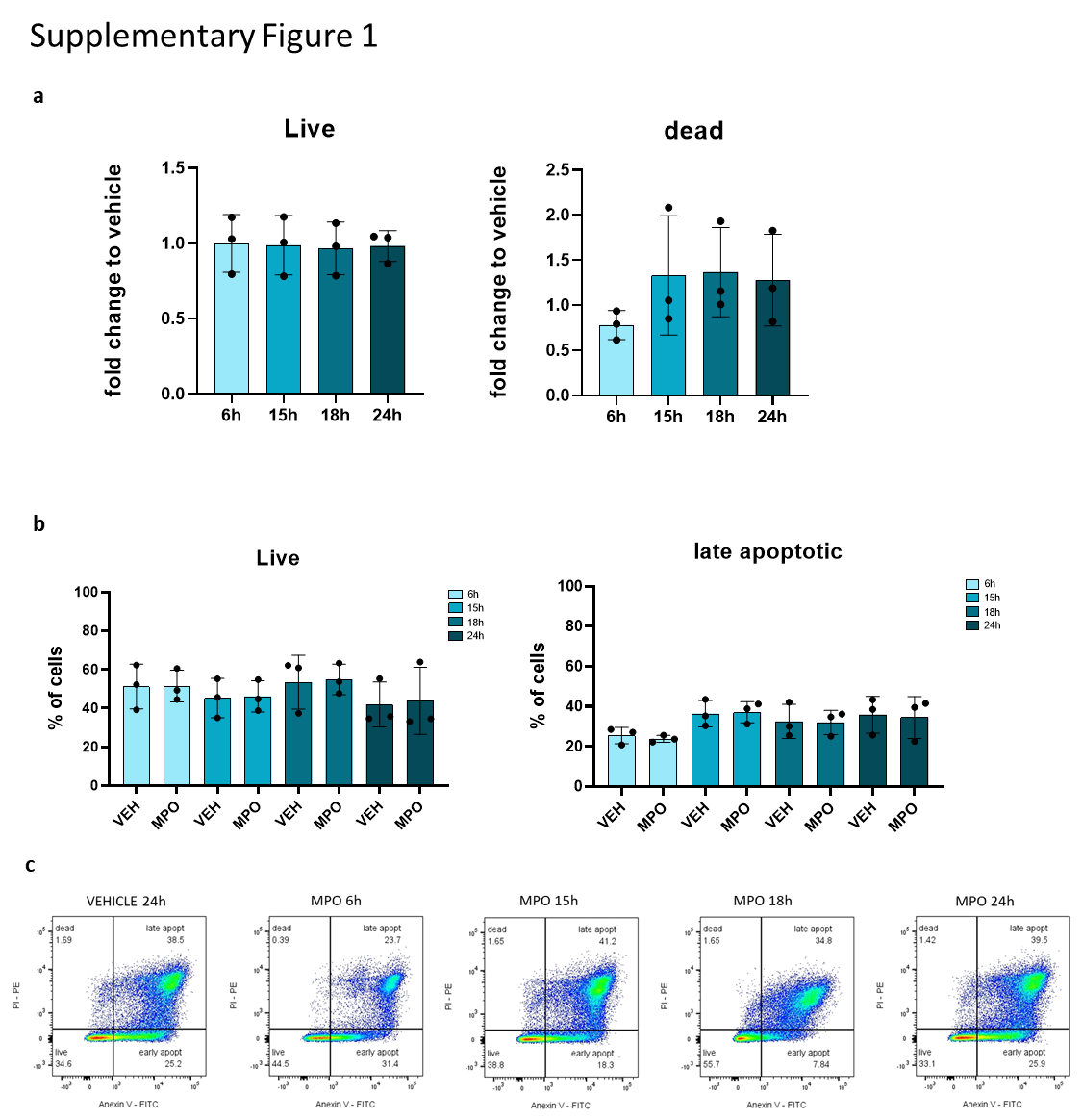
**

**Supplementary Figure 1. JEG-3 apoptosis measurement**

**a)** JEG-3 Annexin V/PI apoptosis assay. Live, and dead cells were measured by flow cytometry following treatment with 5 µg/ml MPO at 6 h, 15 h, 18 h, and 24 h compared with vehicle-treated cells (N = 3). **b)** Live and late apoptotic cells represented as % of all cells upon treatment with 5 µg/ml MPO. Annexin V and PI were measured at 6 h, 15 h, 18 h, and 24 h following MPO treatment and compared with vehicle-treated cells (N = 3). **c)** Representative flow cytometry blots for each time point. One-way ANOVA and Tukey’s post hoc test were performed for multiple comparisons. Data are presented as the mean ± SD, *P < 0.05.


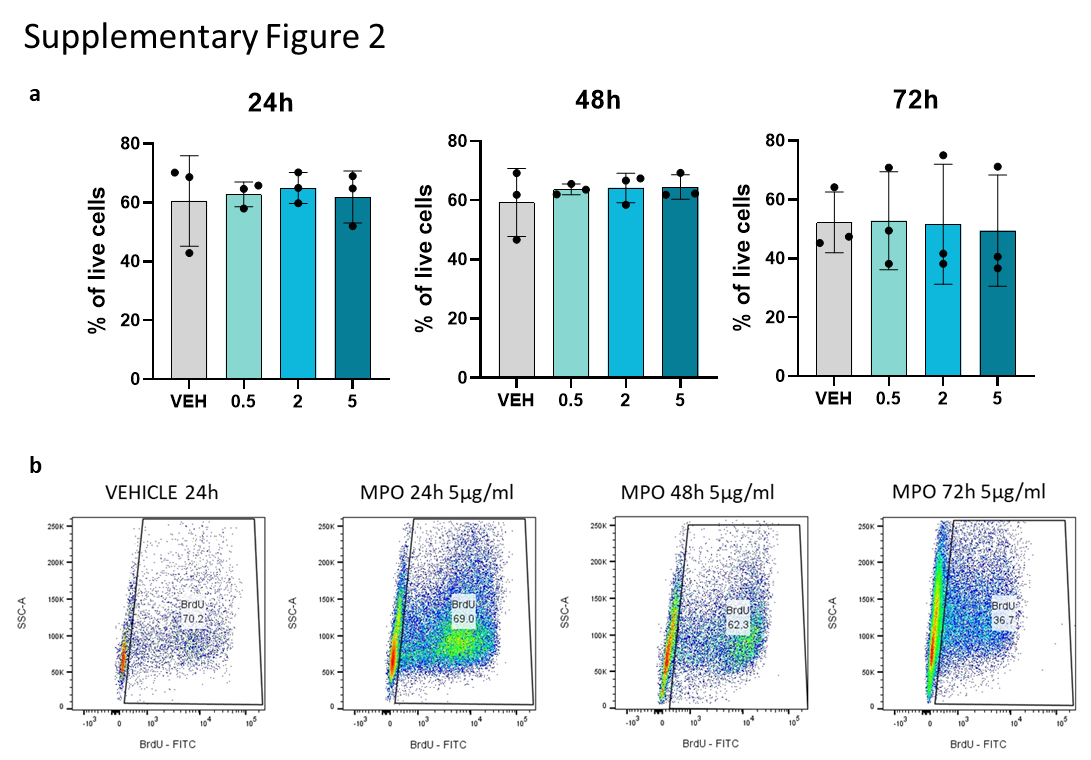


**Supplementary Figure 2. JEG-3 proliferation**

**a)** JEG-3 BrdU assay. BrdU was measured by flow cytometry at 24 h, 48 h, and 72 h following 5 µg/ml MPO treatment. Data is represented as % of live cells (N = 3). **b)** Representative flow cytometry blots for each time point. One-way ANOVA and Tukey’s post hoc test were performed for multiple comparisons. Data are presented as the mean ± SD, *P < 0.05.

**
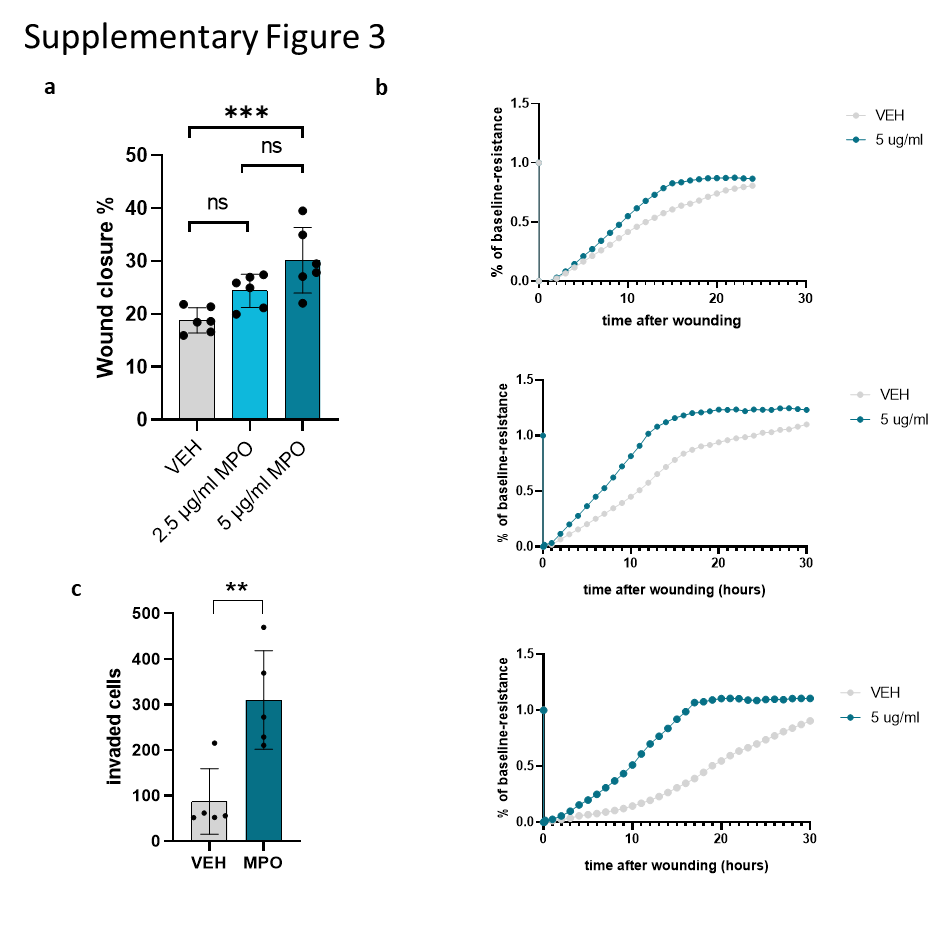
**

**Supplementary Figure 3. MPO induced EVT migration and invasion**

**a)** Scratch assay results are presented as % of wound closure (N = 6). **b)** Results of three ECIS measurements represented as % of baseline resistance through 24 h comparing untreated and MPO treated JEG-3 cells. **c)** Invasion results represented as number of invaded cells (N = 5). One-way ANOVA and Tukey’s post hoc test were performed for multiple comparisons. To compare two samples, an unpaired t-test was performed. Data are presented as the mean ± SD, *P < 0.05.

**
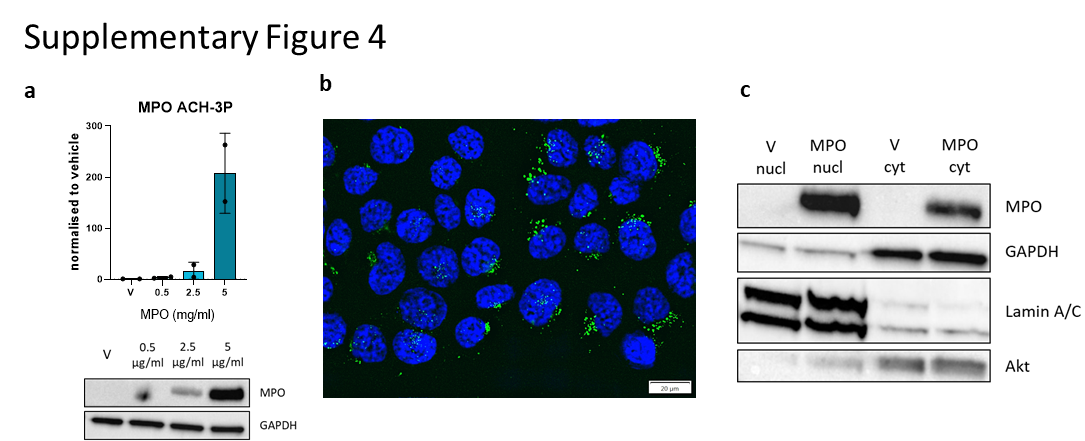
**

**Supplementary Figure 4. MPO uptake and localization in ACH-3P cells**

**a)** Western blot analysis of ACH-3P cells treated with various concentrations of MPO (0.5 µg/ml, 2,5 µg/ml and 5 µg/ml) presented as fold-change relative to vehicle (N = 2). **b)** Representative immunofluorescence microscopy image of 10 µg/ml MPO-treated cells. The cell nucleus was stained with DAPI and MPO is represented in green with AF488-labeled secondary antibody. **c)** Representative fractionation western blot images of MPO, GAPDH, Lamin A/C, and tAKT for vehicle and 5 µg/ml MPO-treated cells. Cytoplasmic and nuclear fractions were isolated. One-way ANOVA and Tukey’s post hoc test were performed for multiple comparisons. Data are presented as the mean ± SD, *P < 0.05.


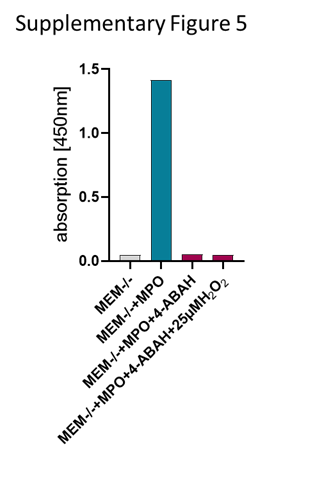


**Supplementary Figure 5. Activity of MPO upon inhibition with 4-ABAH**

MPO activity is blocked in the presence of 4-ABAH (N=1).
